# Development of a quick dot blot assay for the titering of bovine ephemeral fever virus

**DOI:** 10.1186/s12917-019-2059-6

**Published:** 2019-09-02

**Authors:** Li-Ting Cheng, Yu-Jing Zeng, Chun-Yen Chu, Hsian-Yu Wang

**Affiliations:** Graduate Institute of Animal Vaccine Technology, College of Veterinary Medicine, National Pingtung University of Science and Technology, No. 1, Shuehfu Road, Neipu, Pingtung 91201 Taiwan, Republic of China

**Keywords:** Bovine ephemeral fever virus, Dot blot assay, G protein, N protein

## Abstract

**Background:**

Bovine ephemeral fever virus (BEFV) causes fever and muscle stiffness in cattle, resulting in negative economic impact for cattle and dairy farms. During the manufacturing process of inactivated vaccine for virus control, it is important to determine the virus titer, but traditional methods such as plaque assay and TCID_50_ assay require days of waiting time. We sought to develop a quick dot blot assay for BEFV titering.

**Results:**

Three different kinds of BEFV antigens were prepared to raise primary antibodies for BEFV detection in dot blot assays: 1) purified BEFV particles, 2) *Escherichia coli* (*E. coli*)-expressed BEFV G1 region, and 3) *E. coli*-expressed BEFV N protein. Results showed that antibodies raised against purified BEFV particles can detect BEFV particles, but it also showed a high background level from the proteins of BHK-21 cells. Antibodies raised against *E.coli*-expressed BEFV G1 region could not detect BEFV particles in dot blot assays. Finally, antibodies raised against *E.coli*-expressed BEFV N protein detected BEFV particles with a high signal-to-noise ratio in dot blot assays.

**Conclusions:**

*E.coli*-expressed N protein is a suitable antigen for the production of antiserum that can detect BEFV particles with a high signal-to-noise ratio. A dot blot assay kit using this antiserum can be developed as a quick and economical way for BEFV titering.

**Electronic supplementary material:**

The online version of this article (10.1186/s12917-019-2059-6) contains supplementary material, which is available to authorized users.

## Background

Bovine ephemeral fever virus (BEFV) infects cattle and water buffalos through arthropod vectors, causing fever, muscle stiffness, and ocular/nasal discharge. While mortality due to BEFV infection is low, reduced milk production during pandemic periods can result in significant economic losses for dairy farms [1]. As a member of the genus *Ephemerovirus* in the family *Rhabdoviridae*, BEFV has a single-stranded, negative sense RNA genome that encodes five structural proteins (N, P, M, G, and L) and five nonstructural proteins [2–5]. N is the nucleoprotein and G is the envelope glycoprotein, a major antigen that elicits neutralizing antibodies. G contains four antigenic regions (G1-G4), and G1 is a major region of neutralizing epitopes [6].

Inactivated BEFV vaccines serve as the major tool for BEF control. However, antibody titers elicited by inactivated BEFV vaccines are not long-lasting [7, 8] and booster vaccination is required every year, creating a stable market demand for the vaccine.

During the manufacturing process of inactivated BEFV vaccine, a quick and reliable virus titering method is essential. Conventional methods such as TCID_50_ and plaque assays are time-consuming and require days of waiting time. Therefore, the present study aimed to develop a dot blot assay for quick quantitation of BEFV particles in a cell culture preparation. To determine the type of antigens that is most suitable for raising antibodies to be used in dot blot assays, three different kinds of BEFV antigens were prepared: 1) purified BEFV particles, 2) *E. coli*-expressed BEFV G1 region, and 3) *E. coli*-expressed BEFV N protein. Antibodies raised using these antigens were evaluated in dot blot assays.

## Results

### Antibodies raised against purified BEFV particles showed a high background level in dot blot assays

**Fig. 1:**
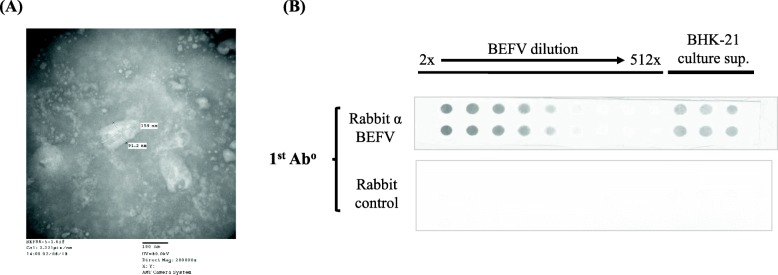
Dot blot assay prepared using antibodies raised against purified BEFV particles. **a** To immunize rabbits for antiserum production, BEFV particles were isolated by the sucrose gradient centrifugation method and imaged by TEM at 50,000x magnification. **b** Dot blot assays were set up with raised rabbit anti-BEFV or negative control antiserum as primary antibodies. Two-fold serial dilutions of BEFV test samples, or BHK-21 cell supernatants, were applied for BEFV detection

To construct a dot blot assay for BEFV quantitation, we first evaluated using purified BEFV particles as the antigen for primary antibody production. BEFV particles cultured in BHK-21 cells were purified by centrifugation in a sucrose gradient. Typical bullet-like virus particles can be observed by transmission electron microscopy (Fig. 1a). Rabbits were then immunized with the purified particles, and the antiserum produced was used as the primary antibody to construct a dot blot assay. When tested using the supernatant of a BEFV culture preparation, the dot blot assay showed signals for virus detection (Fig. 1b upper panel). However, the assay also showed a strong reaction with the supernatant of BHK-21-only cell culture, indicating a high background level (Fig. 1b upper panel). This high noise level makes the rabbit antiserum unusable in a dot blot assay.

### Antibodies raised against *E. coli*-expressed BEFV G1 region cannot detect BEFV particles in dot blot assays

**Fig. 2:**
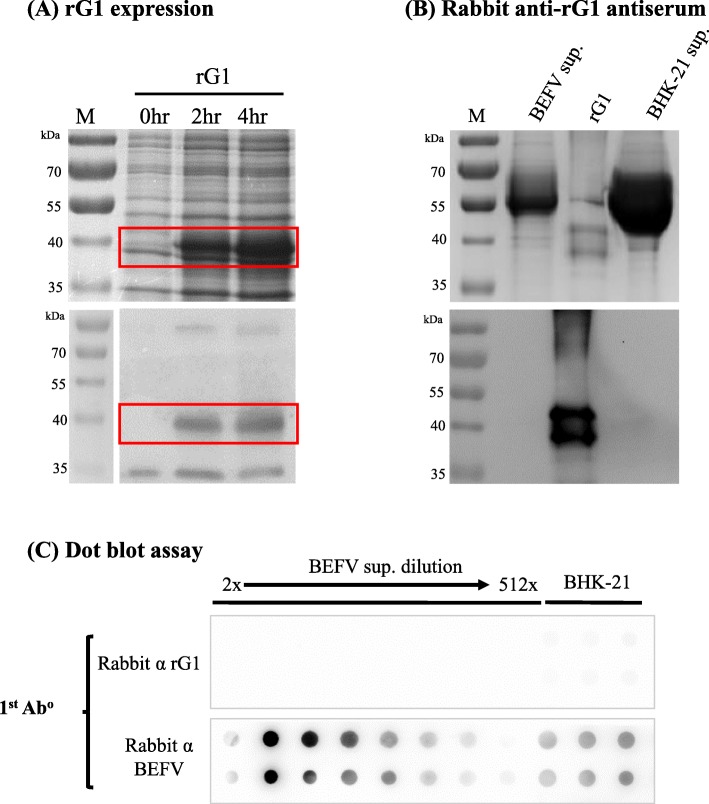
Dot blot assay prepared using antibodies raised against *E. coli*-expressed BEFV G1 region. **a** Recombinant G1 protein expression was induced for 0, 2, and 4 h in *E. coli* and analyzed by SDS-PAGE (upper panel). Rabbit anti-BEFV antiserum was employed to identify the 42 kDa rG1 protein (lower panel). **b** Protein samples of BEFV culture supernatant (BEFV sup.), rG1 protein (rG1), and BHK-21 cell culture supernatant (BHK-21 sup.) were separated by SDS-PAGE (upper panel) and probed by rabbit antiserum raised against the *E. coli*-expressed BEFV G1 region (lower panel). **c** Dot blot assays were set up with raised rabbit anti-rG1 or anti-BEFV antiserum as primary antibodies. Two-fold serial dilutions of BEFV test samples, or BHK-21 cell supernatant, were applied for BEFV detection

To overcome the problem of high background levels, we sought to produce antiserum with BEFV antigens from non-BHK-21 cell culture systems. Using the prokaryotic *E. coli* expression system, the BEFV G1 region was expressed for antiserum production (Fig. 2a upper panel). The estimated molecular weight of the recombinant G1 protein (rG1) was 42 kDa, and the antigenicity of rG1 was confirmed by Western-blotting using the previously produced antiserum raised against purified BEFV particles (Fig. 2a upper panel). Rabbits were then immunized with rG1. When the antiserum produced was first evaluated by Western blotting, while it reacted with rG1, the antiserum, unexpectedly, produced no signals against BEFV particles (Fig. 2b lower panel). Dot blot assays were set up with the antiserum, and consistent with Western blot results, the antiserum cannot recognize BEFV particles (Fig. 2c). Thus, when using *E. coli*-expressed rG1, even though the background level was successfully reduced, we have also lost the signals for BEFV.

### Antibodies raised against *E. coli*-expressed BEFV N protein can detect BEFV particles with a high signal-to-noise ratio in dot blot assays

**Fig. 3:**
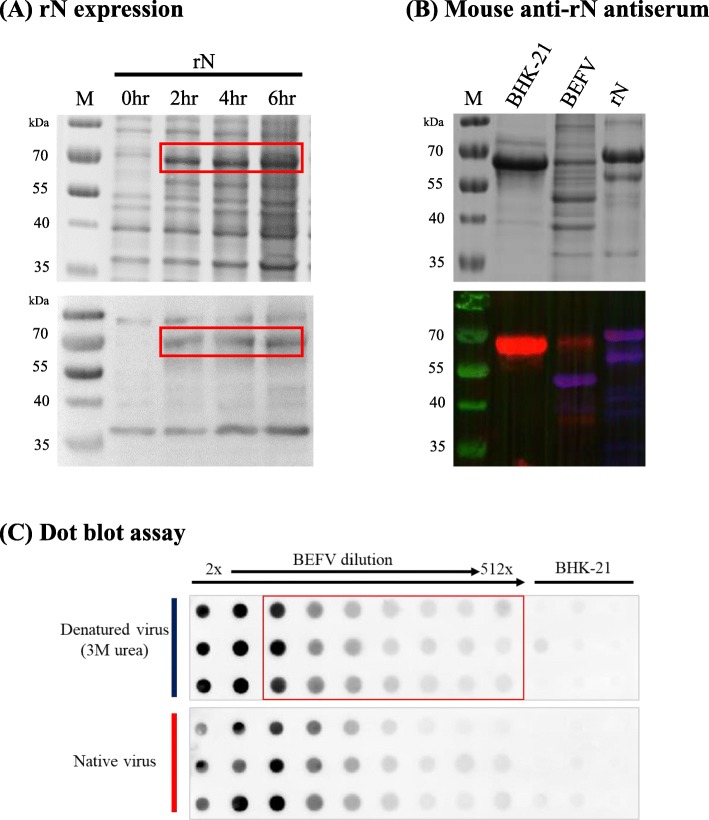
Dot blot assay prepared using antibodies raised against *E. coli*-expressed BEFV N protein. **a** Recombinant N protein (68 kDa) expression was induced for 0, 2, 4, and 6 h in *E. coli* and analyzed by SDS-PAGE (upper panel). Rabbit anti-BEFV antiserum was employed to identify rN protein (lower panel). **b** Protein samples of BHK-21 cell culture supernatant (BHK-21 sup.), purified BEFV (BEFV), and rN protein (rN) were separated by SDS-PAGE (upper panel). In the lower panel, Western blotting was then performed and the samples were co-probed by two different antibodies: rabbit anti-BEFV antiserum (red color), and mouse anti-rN antiserum (blue color). **c** Dot blot assays were set up with raised rabbit anti-rN antiserum as the primary antibody. Two-fold serial dilutions of denatured (upper panel) or native (lower panel) BEFV test samples, or BHK-21 cell supernatant, were applied for BEFV detection

We turned to express an alternative BEFV antigen, the N protein, for antiserum production. Recombinant N protein (rN) was successfully expressed by the *E. coli* expression system (Fig. 3a upper panel), and its antigenicity confirmed (Fig. 3a lower panel). Mice were then immunized with rN and the antiserum produced was evaluated by Western blotting. Results showed that the antiserum can recognize rN and also the N protein (at the 42 kDa position in the BEFV lane, blue florescence) in the BEFV particles (Fig. 3b lower panel). Dot blot assays were set up with the antiserum, and when denatured BEFV particle preparations were applied, clear signals can be observed (Fig. 3c upper panel). Furthermore, background signals from BHK-21 cell culture were minimal. Non-denatured BEFV preparations can also be applied to the dot blot assay, giving similar, albeit less uniform, results (Fig. 3c lower panel). In conclusion, antiserum against *E. coli*-expressed rN can detect cell-cultured BEFV particles with high signal-to-noise ratio in dot blot assays.

**Fig. 4:**
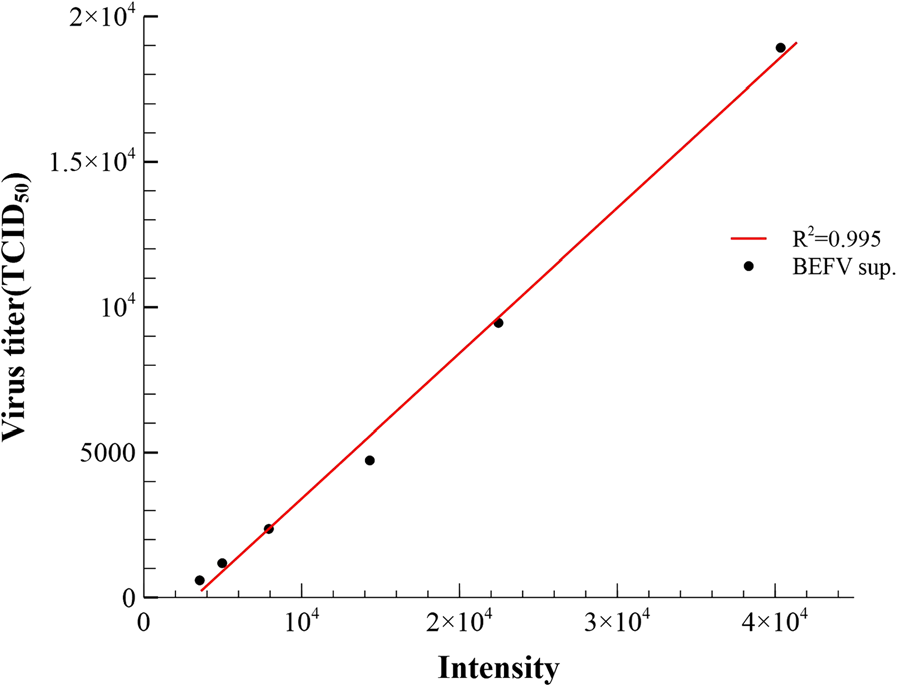
Correlation between TCID_50_ of BEFV and signal intensity measurements from dot blot assay. Dot blot assay using anti-rN antiserum was performed. Signal intensity of 8 to 512x dilution of denatured BEFV in dot blot assay was measured and plotted against corresponding TCID_50_ titers. Calculated R^2^ is 0.995

Since most vaccine manufacturers indicate BEFV titers using TCID_50_, a correlation between TCID_50_ and dot blot assay quantitation was performed, showing strong correlation and convertibility between the two assays (Fig. 4).

## Discussion

When successfully developed, the dot blot method for virus titering is both time-saving and economical. Dot blot assays can be performed in less than 2 h, in contrast to the days that are required for TCID_50_ and plaque assays. In terms of reagent costs, the dot blot method is one of the most economical, with the only main component being a specific primary antibody. The closest type of detection method is the sandwich ELISA method, which exceeds the dot blot method in terms of sensitivity and specificity, but two primary antibodies will be required. For the purpose of virus titering, the dot blot method can provide usable data at minimal cost.

In our study, when whole BEFV particles purified by sucrose gradient were used for antibody production, a high background level was observed in dot blot assays. Residual host cell proteins and serum used in the culturing media may all contribute to the background noise. Further purification of the virus particles could potentially reduce the background noise. Alternatively, affinity columns could be used to purify the antibody, eliminating the anti-host cell antibodies. However, further purification of either the virus or the resulting antibody would also mean increased production cost of the dot blot assay.

In our attempts to produce a working primary antibody for the dot blot assay, G1 appeared to be a suitable antigen for antiserum production. Other groups have shown that BEFV antiserum can recognize *E. coli*-expressed G1 [9]. Therefore, it came as a surprise that the reverse is not true, i.e., antiserum produced against rG1 cannot recognize BEFV particles. This led us to speculate that G1 has both linear epitopes and conformational B-cell epitopes. We further postulate that *E. coli*-expressed G1 retains only the linear epitopes, and thus the antiserum produced against *E. coli*-expressed G1 cannot recognize the conformational epitopes of G1 in a properly folded G protein of BEFV particles. This may have implications for the study of the G1 antigenic region.

The second BEFV antigen that we expressed in *E. coli*, the N protein, is one of the most abundant proteins in the BEFV particle. However, since N is a nucleoprotein that binds to viral RNA, it is found inside the virus particle, making its recognition by antiserum uncertain. Fortunately, results showed that, even without prior treatment, native BEFV particles can be recognized by antiserum raised against rN. The N proteins of BEFV particles are somehow exposed in the assay. To further improve the detection of N protein, a denaturing reagent can be added to the BEFV particles, and more consistent signals can be obtained. In a developed dot blot assay kit, the denaturing reagent can simply be a part of the formulation of the dilution buffer for test samples.

## Conclusion

We found *E. coli*-expressed N protein to be a suitable antigen to produce antiserum that can detect BEFV particles with a high signal-to-noise ratio. Using this antiserum, a dot blot assay kit can be developed as a quick and economical way for BEFV titering.

## Methods

### Cells and virus

For BEFV propagation, Baby Hamster Kidney-21 cells (BHK-21, BCRC#60041, purchased from the Bioresource Collection and Research Center, Taiwan, Republic of China (R.O.C.)) were cultured in Eagle’s Minimum Essential Medium (MEM, Invitrogen, NY, USA) supplemented with 10% fetal bovine serum (FBS, Biological Industries, BH, Israel), at 37 °C in an atmosphere of 5% CO_2_.

BEFV/TW-CYC, an isolate from Taiwan [2], was cultured in BHK-21 cells and virus titers were determined by the Reed and Muench method (1938) and expressed as TCID_50_/mL. Briefly, viruses were serially 10-fold diluted and applied to wells containing 2 × 10^4^ cells/well in 96-well micro-titration plates. The virus stocks were titrated as a positive control while MEM served as a negative control. The plates were incubated at 37 °C, 5% CO_2_ for at least 3 days and observed daily for cytopathic effect.

### Sucrose density gradient ultracentrifugation

To purify BEFV particles, supernatant of BEFV-infected BHK-21 cells was centrifuged at 6000 rpm for 10 min to remove cell debris. Virus particles in the supernatant were first concentrated by centrifugation at 40,000 rpm for 4 h at 4 °C (Himac S50A rotor). After removing the supernatant, virus particles in the pellet were resuspended in a small volume of Phosphate Saline Buffer (PBS) and applied to a 30, 40 and 50% (w/v) sucrose gradient in PBS for centrifugation at 40,000 rpm, 4 °C, for 4 h (Himac S50ST rotor) (Additional file 1: Figure S1). The layer of virus particle was then concentrated, harvested, resuspended in the PBS solution, and stored at -80 °C for further use.

### Recombinant G1 and N expression plasmids

The coding sequence of the G1 region of the BEFV G gene (nucleotides 988 to 1593) was obtained (GenBank accession number: AF208840), optimized for protein expression in *E. coli*, and sent for DNA synthesis (Mission Biotech, Taiwan, R.O.C.). Synthesized gene was then sub-cloned into the pET-32a plasmid (Invitrogen, CA, USA) for protein expression in the *E. coli* strain BL21 (DE3) (Invitrogen, CA, USA). The same gene construction and cloning processes were repeated for the N gene of BEFV (GenBank accession number: AF234533.1, nucleotides 1 to 1284).

### Recombinant protein expression and purification

To express recombinant G1 (rG1) and N (rN) proteins, transformed BL21 (DE3) cells were induced with 1 mM Isopropylthio-β-d-thiogalactose (IPTG) (Amresco, OH, USA) in 37 °C shaking culture. SDS-PAGE and Western blot analyses were performed to confirm protein expression. Briefly, rG1 and rN proteins were analyzed by 10% sodium dodecyl sulfate-polyacrylamide gel electrophoresis (SDS-PAGE) and stained by 0.1% Coomassie blue. For Western blot assays, after gel electrophoresis, protein samples were transferred onto polyvinylidene difluoride (PVDF) membranes (Amersham biosciences, Buckinghamshire, UK), which were then blocked with Hyblock Blocking Buffer (GOALBIO, Taiwan, R.O.C.). As primary antibodies, produced rabbit or mice antisera at 1:1000 dilution were added for incubation at 37 °C for 1 h. Goat anti-rabbit IgG-HRP or goat anti-mouse IgG-HRP at 1:10,000 dilution were used as secondary antibodies. ECL plus Western Blot Detection Reagents (GE Healthcare, WI, USA) were used according to the manufacturer’s instructions. For the multi-color Western blot assay described in Fig. 3b, rabbit anti-BEFV (1:300) and mouse anti-rN antiserum (1:500) were used as the primary antibody. Two different second antibodies, Goat anti-rabbit IgG(H + L) HRP conjugate (1:3000) (#1706515, BIO-RAD, California, USA) and StarBrightTM Blue 700 Goat anti-mouse IgG (1:3000) (#12004158, BIO-RAD, California, USA) were used as the secondary antibodies. The image was captured using the ChemiDoc™ MP Imaging System (BIO-RAD, California, USA).

For protein purification, induced BL21(DE3) cells were pelleted, resuspended in denaturing lysis buffer (6 M Urea), sonicated using Misonix Sonicator S-4000 (Misonix, New York, USA), and passed through 0.45 μm filters. rG1 and rN were then purified using the ProfiniaTM Protein Purification System (BIO-RAD, California, USA) with histidine affinity column (bio-ScaleTM Mini Profinity TM IMAC Cartridge) according to the manufacturer’s instructions. After protein elution, urea in protein samples was gradually dialyzed out by 6 M, 3 M, 1 M urea and pure water. Protein concentration was determined with SDS-PAGE analysis using BSA standards.

### Antibody production

Rabbits were used for anti-whole BEFV and anti-rG1 antibody production. Antigens (50 μg of purified BEFV particles or 50 μg of purified rG1 protein) were formulated with the oil adjuvant ISA206 (SEPPIC, Paris, France) at the suggested ratios. For negative control, PBS was also formulated with ISA206. Three (1 for each vaccine) healthy New Zealand white rabbits (2~3 kg, purchase from the Livestock Research Institute, Taiwan, R.O.C.) were subcutaneously immunized with 1 mL of the prepared vaccines. The rabbits were then boosted by the same vaccines on day 14 and were sacrificed on day 28 for all anti-sera collection. To sacrifice the rabbits, general anesthesia was induced by intramuscularly injection of Zoletil (18 mg/kg, Virbac, Carros, France) and Rompun (0.05 ml/kg, Bayer, Leverkusen, Germany), followed by heart puncture for exsanguination for blood collection.

For anti-rN antibody production, 50 μg of the purified rN protein was formulated with ISA206 and five BALB/c mice (LASCO, Taiwan, R.O.C.) were immunized intraperitoneally (0.2 ml/mice). As the negative control, five BALB/c mice were intraperitoneal injected (0.2 ml/mice) with PBS-ISA206 vaccine. The mice were then boosted by the same vaccines on day 14 and were sacrificed on day 28 for all anti-sera collection. To sacrifice the mice, general anesthesia was induced by intraperitoneal injection of Zoletil (0.5 mg/10 g), followed by heart puncture for exsanguination for blood collection.

### Dot blot assay

To set up a dot blot assay, supernatant of BEFV-infected BHK-21 cells was 2-fold serially diluted with PBS and blotted onto nitrocellulose membrane by suction through the dot-blotting equipment GFE9600 (Bio-East Technology, Taiwan, R.O.C.). For negative control, supernatant of BHK-21 cells was used. The membrane was then blocked with the Hyblock Blocking Buffer (GOALBIO, Taiwan, R.O.C.) for 1 min at room temperature. Three washes with PBST (PBS with 0.05% Tween-20) were performed and the membrane was probed for 1 h with the indicated primary antibody at the following dilutions: 1000x for rabbit anti-whole BEFV antibody, 1000x for rabbit anti-rG1 antibody, and 750x for mouse anti-rN antibody. After another three washes with PBST, the membrane was incubated with the secondary antibody: either goat anti-rabbit IgG-HRP (1:10,000) or goat anti-mouse IgG-HRP (1:5000) in PBST containing 0.5% skim milk, for 1 h at 37 °C. The membrane was then washed again before fluorescence signal development using the Immobilon^®^ Crescendo Western HRP Substrate (MILLPORE, MA, USA). Image of the blot was captured by Fusion Solo (Vilber, Paris, France) for quantitation by ImageJ (v.1.52). Signals of test samples were determined and shown as intensity values as previously described [10, 11]. Calculations and linear regression were performed using the Microsoft Excel program.

## Additional file

Additional file 1: **Figure S1.** BEFV virus particle purification using sucrose gradient centrifugation. (PPTX 2074 kb)

## Data Availability

The datasets used and analyzed during the current study are available from the corresponding author on reasonable request.

## Abbreviations

BEFV sup: BEFV culture supernatant
BEFV: Bovine ephemeral fever virus
BHK-21 sup: BHK-21 cell culture supernatant
FBS: Fetal bovine serum
IPTG: Isopropylthio-β-d-thiogalactose
MEM: Eagle’s Minimum Essential Medium
PBST: PBS with 0.05% Tween-20
PVDF: polyvinylidene difluoride
rG1: recombinant BEFV G1 protein
rN: recombinant BEFV N protein

## References

[1] Ting LJ, Lee MS, Lin YL, Cheng MC, Lee F (2016). Invasion of exotic bovine ephemeral fever virus into Taiwan in 2013-2014. Vet Microbiol.

[2] Hsieh YC, Wang SY, Lee YF, Chen SH, Mak POT, Chu CY (2006). DNA sequence analysis of glycoprotein G gene of bovie ephemeral fever virus and development of a double oil emulsion vaccine against bovine ephemeral fever. J Vet Med Sci.

[3] Chung YC, Shen HY, Cheng LT, Liu SS, Chu CY (2016). Effectiveness of a BHV-1/BEFV bivalent vaccine against bovine herpesvirus type 1 infection in cattle. Res Vet Sci.

[4] Zheng F, Qiu C. Phylogenetic relationships of the glycoprotein gene of bovine ephemeral fever virus isolated from mainland China, Taiwan, Japan, Turkey, Israel and Australia. Virol J. 2012;9:268. 10.1186/1743-422X-9-268 PMC3502394 23150932

[5] Walker PJ, Klement E. Epidemiology and control of bovine ephemeral fever. Vet Res. 2015;46:124. 10.1186/s13567-015-0262-4 PMC4624662 26511615

[6] Cybinski DH, Kongsuwan K, Walker PJ, Cooper J (2015). Location of neutralizing epitopes on the G protein of bovine ephemeral fever rhabdovirus. J Gen Virol.

[7] Tzipori S, Spradbrow PB (1978). A cell culture vaccine against bovine ephemeral fever. Aust Vet J.

[8] Zheng FY, Chen QW, Li Z, Gong XW, Wang JD, Yin H (2016). Experimental infection with bovine ephemeral fever virus and analysis of its antibody response cattle. Res Vet Sci.

[9] Yazdani F, bakhshesh M, Esmaelizad M, Sadigh ZA (2017). Expression of G1- epitope of bovine ephemeral fever virus in E. coli : a novel candidate to develop ELISA kit. Vet Res Forum.

[10] Heidebrecht F, Heidebrecht A, Schulz I, Behrens SE, Bader A (2009). Improved semiquantitative Western blot technique with increased quantification range. J Immunol Methods.

[11] Guo HC, Jin Y, Han SC, Sun SQ, Wei YQ, Liu XJ (2015). Quantitative proteomic analysis of BHK-21 cells infected with foot-and-mouth disease virus serotype Asia 1. PLoS One.

