# Supplementary material for: Development of a quick dot blot assay for the titering of bovine ephemeral fever virus
Source: BMC Vet Res. 2019 Sep 2;15:313. doi: 10.1186/s12917-019-2059-6 (PMC6720828; doi:10.1186/s12917-019-2059-6)
Supplement: Supplementary file 1 — Figure S1. BEFV virus particle purification using sucrose gradient centrifugation. (PPTX 2074 kb) [file 12917_2019_2059_MOESM1_ESM.pptx]

## Slide 1
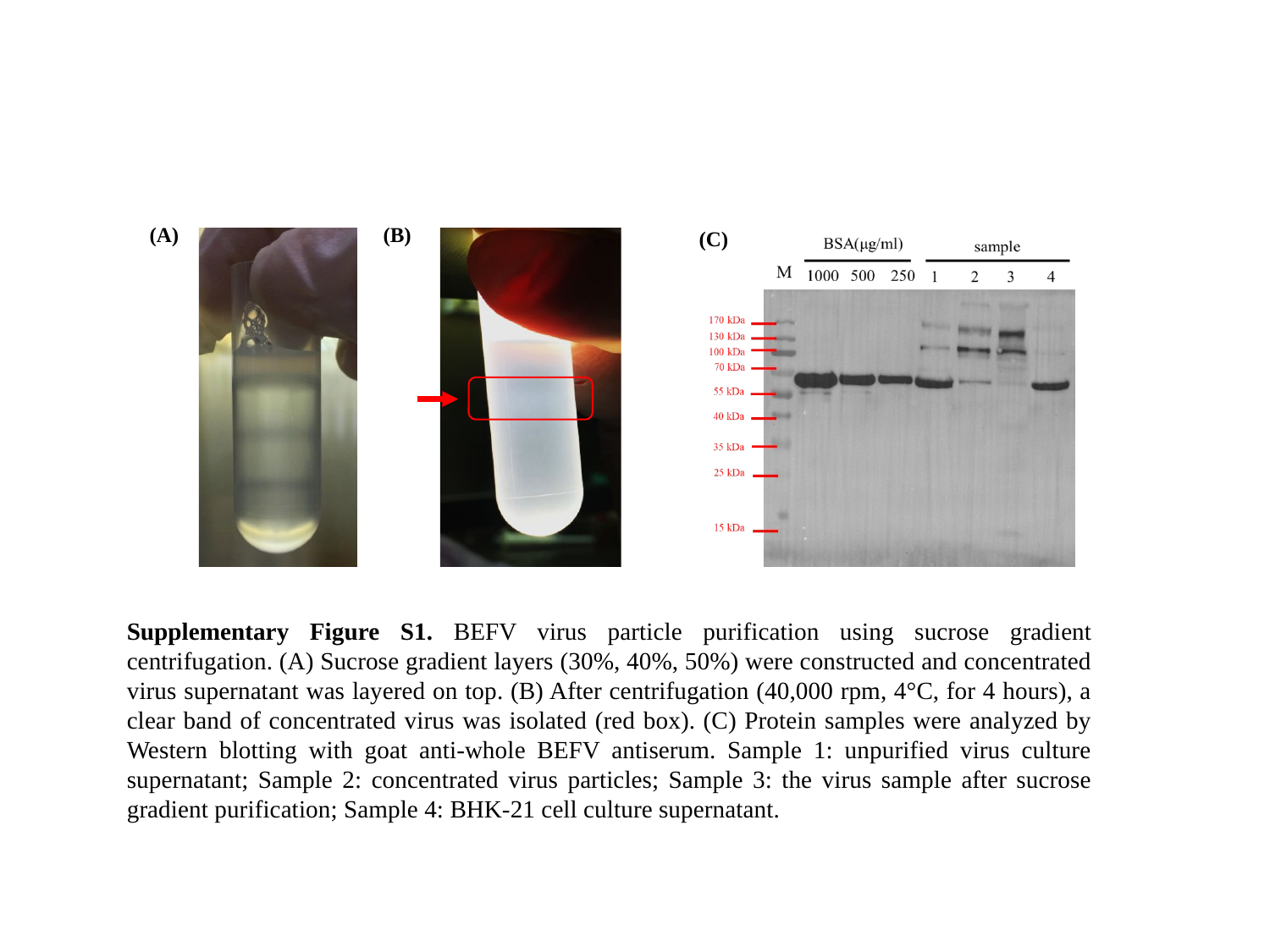

(A)
(B)
(C)
Supplementary Figure S1. BEFV virus particle purification using sucrose gradient centrifugation. (A) Sucrose gradient layers (30%, 40%, 50%) were constructed and concentrated virus supernatant was layered on top. (B) After centrifugation (40,000 rpm, 4°C, for 4 hours), a clear band of concentrated virus was isolated (red box). (C) Protein samples were analyzed by Western blotting with goat anti-whole BEFV antiserum. Sample 1: unpurified virus culture supernatant; Sample 2: concentrated virus particles; Sample 3: the virus sample after sucrose gradient purification; Sample 4: BHK-21 cell culture supernatant.
